# The impact of biological sex on the response to noise and otoprotective therapies against acoustic injury in mice

**DOI:** 10.1186/s13293-018-0171-0

**Published:** 2018-03-12

**Authors:** Béatrice Milon, Sunayana Mitra, Yang Song, Zachary Margulies, Ryan Casserly, Virginia Drake, Jessica A. Mong, Didier A. Depireux, Ronna Hertzano

**Affiliations:** 1 Department of Otorhinolaryngology - Head and Neck Surgery, University of Maryland, 16 South Eutaw Street, Suite 500, Baltimore, MD 21201 USA; 2 Institute for Genome Science, University of Maryland School of Medicine, Baltimore, MD 21201 USA; 3 Department of Pharmacology, University of Maryland School of Medicine, Baltimore, MD 21201 USA; 4 Institute for Systems Research, University of Maryland, College Park, MD 20742 USA; 5 Department of Anatomy and Neurobiology, University of Maryland School of Medicine, Baltimore, MD 21201 USA

**Keywords:** Noise-induced hearing loss, Sex differences, SAHA, B6CBAF1/J mice, Inner ear, ABR

## Abstract

**Background:**

Noise-induced hearing loss (NIHL) is the most prevalent form of acquired hearing loss and affects about 40 million US adults. Among the suggested therapeutics tested in rodents, suberoylanilide hydroxamic acid (SAHA) has been shown to be otoprotective from NIHL; however, these results were limited to male mice.

**Methods:**

Here we tested the effect of SAHA on the hearing of 10-week-old B6CBAF1/J mice of both sexes, which were exposed to 2 h of octave-band noise (101 dB SPL centered at 11.3 kHz). Hearing was assessed by measuring auditory brainstem responses (ABR) at 8, 16, 24, and 32 kHz, 1 week before, as well as at 24 h and 15–21 days following exposure (baseline, compound threshold shift (CTS) and permanent threshold shift (PTS), respectively), followed by histologic analyses.

**Results:**

We found significant differences in the CTS and PTS of the control (vehicle injected) mice to noise, where females had a significantly smaller CTS at 16 and 24 kHz (*p* < 0.0001) and PTS at 16, 24, and 32 kHz (16 and 24 kHz *p* < 0.001, 32 kHz *p* < 0.01). This sexual dimorphic effect could not be explained by a differential loss of sensory cells or synapses but was reflected in the amplitude and amplitude progression of wave I of the ABR, which correlates with outer hair cell (OHC) function. Finally, the frequency of the protective effect of SAHA differed significantly between males (PTS, 24 kHz, *p =* 0*.*002) and females (PTS, 16 kHz, *p =* 0.003), and the magnitude of the protection was smaller in females than in males. Importantly, the magnitude of the protection by SAHA was smaller than the effect of sex as a biological factor in the vehicle-injected mice.

**Conclusions:**

These results indicate that female mice are significantly protected from NIHL in comparison to males and that therapeutics for NIHL may have a different effect in males and females. The data highlight the importance of analyzing NIHL experiments from males and females, separately. Finally, these data also raise the possibility of effectors in the estrogen signaling pathway as novel therapeutics for NIHL.

**Electronic supplementary material:**

The online version of this article (10.1186/s13293-018-0171-0) contains supplementary material, which is available to authorized users.

## Background

Noise-induced hearing loss (NIHL) is a form of an acquired hearing deficit that underlies 16% of adult sensorineural hearing loss worldwide [1]. In the US adult population, NIHL is second only to age-related hearing loss (ARHL) [2]. NIHL as an occupational hazard is widespread in the military, construction, agriculture, and in other fields with high noise exposure, causing hearing loss in 7–21% of the exposed population [3]. Health problems secondary to noise exposure are particularly frequent in the military. In the USA, hearing loss and tinnitus rank as the most prevalent service-connected disabilities for veterans [4]. Untreated hearing loss adversely impacts social, psychological, and cognitive functioning of affected individuals [5].

Small animal models such as the guinea pig, gerbil, chinchilla, mouse, and ferrets are commonly used to conduct auditory research and, in particular, studies on NIHL [6–8]. Mouse models have proven especially useful because of the ease in generating inbred strains with low genetic variability, the ability to manipulate the mouse genome, as well as structural, molecular and functional similarity to the human ear [9–11]. The current study stemmed from research that was designed to analyze the molecular mechanism of action of drugs with a protective effect from NIHL. Suberoylanilide hydroxamic acid (SAHA) is a histone deacetylase inhibitor and thus functions through modulating gene expression by changing the accessibility of the DNA to transcription factors [12, 13]. SAHA has been shown to be protective against hearing loss caused by exposure to chemicals/medications (ototoxic hearing loss) in vivo in mice of both sexes [12] as well as from NIHL in male mice [14]. Little is known, however, about the differential responses of male and female mice to noise and its potential therapeutics as historically most studies of acquired hearing loss using animal model were performed exclusively on males [14–19]. This is in part because the fluctuating hormone levels during an estrous cycle could introduce a confounding variable in the response to trauma or treatment [20]. Of relevance, sex differences have been described in age-related hearing loss (presbycusis) as well as in NIHL, where pre-menopausal women are protected in comparison to age-matched men [21, 22].

Here we initially tested the efficacy of SAHA as a protective agent from NIHL in young adult B6CBAF1/J mice of both sexes. We exposed mice of both sexes, who were treated with SAHA or its carrier, DMSO, to a permanent threshold-shift inducing noise exposure. We compared hearing function by analysis of auditory brainstem responses, and histological outcomes of the noise exposures by inner and outer hair cell counts and inner hair cell synapse analysis. Our results indicate a differential response to both noise and SAHA treatment between sexes, where female mice exhibit less hearing loss following noise (i.e., less damage) and have less therapeutic benefit from SAHA, when compared to males. Interestingly, the effect of sex on the degree of hearing loss following noise exposure was greater than the effect of the tested drug. This is the first detailed report comparing and characterizing such differences between sexes.

## Methods

### Animals

All procedures involving animals were carried out in accordance with the National Institutes of Health Guide for the Care and Use of Laboratory Animals and were approved by the Animal Care Committee at the University of Maryland (protocol numbers 0915006 and 1015003) and the Animal Care and Use Review Office (Department of Defense, USA).

All experiments were performed on B6CBAF1/J mice (Stock No: 100011, Jackson Laboratories, ME). We use B6CBAF1/J mice, which are F1 progeny of a cross between C57BL/6J and CBA/J (CBA) for most of our experiments for NIHL. We choose this combination of strains because, while the C57BL/6 mice are used extensively to generate transgenic animals for auditory research owing to availability of its complete genome information [23], long life span and resistance to sound induced seizures [24, 25]; C57BL/6 mice also suffer from early onset age-related hearing loss (ARHL) due to recessively inherited mutation in the *Cdh23* gene [26] underlying the *Ahl* locus [27]. In contrast, the CBA strain is relatively resistant to ARHL [28]. The B6CBAF1/J mice therefore enable the use of Cre-lines (originating from C57BL/6 mice) and have been previously used and characterized in studies of NIHL [29]. Mice were obtained at 7–8 weeks of age and kept in our facility 1–2 weeks for acclimatization before any procedures. The facility is controlled for temperature and humidity, has a 12h light/12 h dark cycle (lights on at 6 am), and mice were provided with food and water ad libitum.

### Study design

The experiment consisted of two separate cohorts of animals, to ascertain reproducibility of data. Each cohort consisted of (a) three male and three female mice that were not exposed to noise and used only for histology, (b) six or eight mice from each sex that were all exposed to noise and treated with SAHA, and (c) six or eight mice from each sex that were all exposed to noise and treated with vehicle (DMSO). Noise exposures were performed on 3 to 4 mice at a time, which consisted of a mixture of mice that were treated with either SAHA or DMSO. The phase of the estrus cycle was not recorded for the female mice. One male in the DMSO group was removed from all analyses because it did not present a threshold shift at 24 h after noise exposure. A second person who was blinded with respect to the animal groups determined the ABR thresholds and counted outer hair cells and synapses.

### Noise trauma

All noise exposures were performed on mice at 10 weeks of age. Noise trauma was induced with a 2-h duration, octave band of noise centered at 11.3 kHz (8–16 kHz) at 101 dB sound pressure level (SPL) using the Fostex FT17H tweeter [30] (Fostex, Tokyo, Japan). Output stimuli were calibrated with a measurement microphone (PCB Piezoelectronics, NY) placed at the same distance as the mouse ears. Mice were placed in a custom-made animal holder made of a perforated aluminum sheet, 18 × 15 × 5 cm in size with eight equal-sized chambers measuring 4.5 × 7.5 × 5 cm, which was itself placed in a soundproof box (IAC Acoustics, IL). Only the four center chambers immediately inferior to the speaker were used to house mice during the noise exposures. Sound level was measured to be within 0.5 dB of the target level throughout the holding cells, with the speaker situated 20 cm above the mice. The mice were awake and unrestrained throughout the noise exposure. All mice were exposed to noise at the same time of the day (8 am) for each of the experimental groups.

### SAHA treatment

Mice were injected intraperitoneally with suberoylanilide hydroxamic acid (SAHA) (Selleckchem, TX), (100 mg/kg body weight) dissolved in DMSO (MilliporeSigma, MA), or with DMSO alone (vehicle) 3 days before exposure to noise and 2 h after the end of the noise exposure. The SAHA dosing amount was based on a previous publication using SAHA as an otoprotective agent, where the authors tried different dose concentrations of SAHA and reported a 100 mg/kg dose to be most efficacious without cytotoxicity [12]. Because studies vary in their dosing regimen for SAHA, the frequency of the dosing was based on the published literature with minor modifications [14, 31].

### Determination of auditory brainstem response

Auditory brainstem responses (ABR) were recorded after induction of anesthesia using an intraperitoneal injection of a mixture of ketamine (100 mg/kg) (VetOne, ID) and xylazine (20 mg/kg) (Anased, IL). Hearing thresholds were determined at 8, 16, 24, and 32 kHz using the RZ6 recording system (Tucker-Davis Technologies, FL). Recording electrodes were inserted under the skin at the inferior post-auricular area of the left and right ears, and a reference was placed under the skin at the vertex region of the skull. A ground electrode was inserted near the base of the tail. The animals were placed in a soundproof box (IAC Acoustics, IL) for the recordings. Stimuli were presented via a speaker situated in front of the mouse, 10 cm from the ears. Frequency-specific tone bursts 2.5 ms long, with a 0.5 ms sinusoidal on and off ramp, were presented to the mice at varying intensities beginning at 90 dB SPL and proceeding in 5 dB decrements down to 10 dB below the measurable hearing threshold for each mouse. Output stimuli were calibrated with a one-quarter inch microphone (model PCB-378C01; PCB Piezotronics, NY) placed at the same distance from the speaker as the mouse ears would be. Electrophysiological signals in response to each tone stimulus were recorded for 10 ms starting at the onset of the tone. A total of 512 sweeps were presented at the rate of 21 sweeps/s, and responses were averaged at each level and frequency tested. Responses from both ears were recorded simultaneously and used for data acquisition [32]. Hearing thresholds were determined as the lowest level at which definite ABR waves I and II response patterns could be identified for each frequency. Importantly, wave I and II of the ABR are generated from the contributions of the uncrossed fibers of spiral ganglion and cochlear nucleus, respectively. This allowed for hearing thresholds to be determined from both ears simultaneously, with each ear considered a separate data point. The results section shows the data with each ear counting as an individual biological replicate because both ears were exposed to noise and thresholds were obtained from the two ears separately, as previously described [33, 34]. In addition, the supplementary data reports the hearing threshold results where the thresholds from both ears of each mouse are averaged and each mouse is counted as an individual biological sample. Body temperature of the animals was maintained constant at 37.0 °C by a feedback heating pad placed under the animal while recording (FHC, ME). Baseline ABR thresholds were determined 1 week prior to noise exposure when the mice were 9 weeks of age. After the noise exposure, ABR thresholds were recorded at 24 h, 8 days, and 15 to 21 days, corresponding to 10–13 weeks of age. These permitted measurement of the compound threshold shift (CTS) as well as permanent threshold shift (PTS), respectively [35].

### ABR wave I amplitude growth as a function of sound level

Peak-to-trough amplitude values of wave I of the ABR traces were extracted using a custom MatLab (MathWorks, MA) script (Additional file 1). Briefly, the script extracted the first maximum deflection after the first millisecond (ms) of the recording (peak I) and the corresponding subsequent minimum deflection (trough I). Wave I peak-to-trough amplitudes were obtained for stimuli levels ranging from 55 dB SPL to 85 dB SPL for ABR recorded before and after noise exposure. In noise-exposed animals, the minimum hearing threshold averaged around 55 dB at 24 h. Thus, the linear regressions were performed setting the minimal value to 55 dB to allow for the comparison of data from all time points. Additionally, the data between these level ranges are linear for most of the level versus amplitude plot, allowing for the accurate calculation of the slope. The data were plotted to obtain the growth of amplitude as a function of sound level for each experimental group at 16 kHz, which was the frequency with the maximal permanent threshold shift. Linear regression analyses were performed using the Prism 7 software (GraphPad, CA) to obtain slope values. The slopes were compared between conditions at each frequency analyzed. Slopes were considered significantly different if *p* < 0.05 calculated by a two-tailed paired *t* test [36].

### Immunostaining

Within 1 week of the final ABR recording, mice were euthanized by CO_2_ asphyxiation followed by cervical dislocation. Immediately after euthanasia, the temporal bones were dissected in ice-cold phosphate-buffered saline (PBS) (Corning, MA), a small hole was made in the bony apex of the cochlea, and the round and oval windows were opened for subsequent perfusion of the fixative. The temporal bones were fixed overnight at 4 °C in 4% paraformaldehyde (Alfa Aesar, MA) solution in PBS and then decalcified by immersion in 500 mM EDTA at 4 °C until adequate decalcification. Each cochlear duct was dissected according to the method described by the Eaton-Peabody Laboratories [37]. Briefly, each cochlear duct was first bisected across the oval window. The resulting halves were further dissected to obtain the apical turn of the basilar membrane as a single piece, the middle turn and the basal turns in two halves as well as the basal hook as a final piece, exposing the organ of Corti in its entirety. The tissue was permeabilized for 1 h in PBST ((PBS (CorningCellgro, VA) with 0.3% Triton X-100 (MiliporeSigma, MA)) and blocked for 1 h in PBST with 5% normal goat serum (Cell Signaling Technologies, MA) at room temperature.

For pre-synaptic ribbon and post-synaptic density staining, cochlear segments were incubated overnight at 37 °C with a monoclonal mouse anti-CtBP2 antibody (1:200, BD Biosciences, CA) and a monoclonal mouse anti-GluR2 antibody (1:2000, MiliporeSigma), diluted in blocking buffer. Labeling was performed by incubating the tissue with the corresponding secondary antibodies, goat anti-mouse IgG2 Alexa Fluor® 488 and goat anti-mouse IgG1 Alexa Fluor® 568 (1:1000, ThermoFisher Scientific, MA) supplemented with DAPI (1:20,000, ThermoFisher Scientific) in PBST for 2 h at room temperature. Tissue was mounted with the ProLong Gold antifade reagent (ThermoFisher Scientific).

### Frequency-specific pre-synaptic ribbon and post-synaptic density (PSD) counts

Following immunostaining, tissue was imaged at a × 20 magnification using a Nikon Eclipse E600 fluorescence microscope (Nikon, NY) equipped with an Infinity 3 camera (Lumenera, Canada) to allow for frequency mapping. Cochlear frequencies were mapped onto the images using the Measure Line plugin, developed by the Eaton-Peabody Laboratories [38] on the software ImageJ [39]. Subsequently, confocal Z-stacks in the regions of 15 to 17 kHz, 22 to 26 kHz, and 32 kHz were obtained using a 63X oil objective, 1.2 X digital zoom, and 42 μm sections using the LSM 5 Duo confocal microscope (Zeiss). Ribbons and PSDs were counted using the ImageJ Cell Counter plugin.

### Cytocochleograms

Fluorescence images of the outer hair cell nuclei counterstained with DAPI were captured using an Eclipse E600 microscope (20 X objective) (Nikon) equipped with an Infinity 3 camera (Lumenera). Cochlear frequencies were mapped as described above for the ribbon and PSD counts. Missing outer hair cells (OHCs) were counted throughout the entire length of the basilar membrane from the apex towards the base at the following frequency intervals: 4–5.6 kHz, 5.6–8 kHz, 8–11.3 kHz, 11.3–16 kHz, 16–22.6 kHz, 22.6–32 kHz, 32–45.2 kHz, 45.2–51 kHz, and 51–55 kHz. These counts were expressed as the percentage of missing OHCs with respect to their position along the length of the basilar membrane.

### Statistical analysis

The ABR data comparisons between groups were made by a two-way ANOVA followed by a Tukey post hoc test for multiple pair-wise comparisons [40]. ABR data comparing threshold shifts within a group before and after noise exposure were analyzed by a two-way ANOVA with Sidak’s post hoc test for multiple comparisons. An adjusted *p* value of < 0.05 was set as the threshold for significance. The *F* values for main effects are listed in the main text, and the interactions are listed in Additional file 2. The value of Cohen’s *d* (d) was calculated when the data reach significance following the post hoc test. All ABR data analyses and figures were generated using Prism 7 software (GraphPad, CA) with the recommended settings for post hoc tests.

For the ABR wave I amplitude analysis, the growth of the amplitude as a function of stimuli levels was expressed as a slope, obtained by linear regression of amplitude versus stimuli level plots from noise-exposed animals (DMSO treated) of each sex, before and after noise exposure, at 16 kHz. Two-tailed *t* test was used to compare the slopes between groups using Prism 7 software (GraphPad, CA).

Comparisons of OHCs and synapse counts between male and female mice were made using Student’s *t* test assuming unequal variance using Prism 7 software (GraphPad, CA).

## Results

### Differential response of male and female mice to noise trauma

**Fig. 1:**
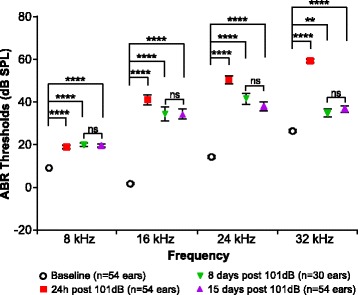
Octave band noise exposure at 101 dB SPL causes PTS in 10-week-old B6CBAF1/J mice. Hearing thresholds were compared between baseline, 24 h, 8 days, and 15 days post-noise exposure. At 24 h post-noise, significant compound threshold shifts are seen across all frequencies tested. Significant permanent threshold shifts are also detected at all frequencies tested at 8 and 15 days post-noise exposure. (**p < 0.01; **** *p* < 0.0001; ns non-significant)

In the present study, 10-week-old male and female mice were exposed to 101 dB SPL octave band noise centered at around 11.3 kHz, for 2 h. The mice received either SAHA (100 mg/kg) dissolved in DMSO, or DMSO alone as a control (vehicle) 3 days before and 2 h after the end of noise exposure. Hearing thresholds were measured using ABRs at 8, 16, 24, and 32 kHz. Thresholds measured at 24 h, 8 days, and 15 days after noise exposure were compared to the baseline thresholds to calculate the compound (24 h) and permanent (8 and 15 days) threshold shifts, respectively (CTS and PTS) [35]. The CTS reflects the change in hearing threshold shortly following noise exposure, which is normally higher than the final change in hearing threshold, whereas the PTS reflects what is considered a “final” change in hearing threshold following noise exposure [35]. In vehicle-treated mice, a two-way ANOVA revealed main effects of frequency and time on the hearing thresholds (frequency: *F*_3, 752_ = 155.1; *p* < 0.0001; time: *F*_3, 752_ = 284; *p* < 0.0001). A post hoc comparison showed that the noise exposure induced a significant CTS and PTS at all frequencies measured (Fig. 1). In addition, there were no statistically significant differences in the hearing thresholds at 8 and 15 days post-exposure; therefore, subsequent measures for PTS are reported at 15 days only (Fig. 1).

**Fig. 2:**
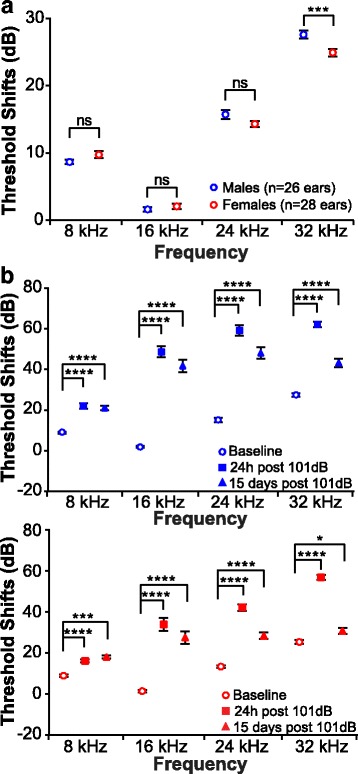
Octave band noise exposure at 101 dB SPL causes PTS in male and female mice. **a** Comparison of baseline hearing threshold between males and females. Female mice present a lower threshold at 32 kHz. **b** Hearing thresholds were compared between baseline, 24 h, and 15 days post-noise exposure in males (top) and females (bottom). At 24 h and 15 days post-noise exposure, significant compound threshold shifts are seen across all frequencies tested in both males and females. (*p < 0.05; ***p < 0.001; **** < 0.0001; ns non-significant)

**Fig. 3:**
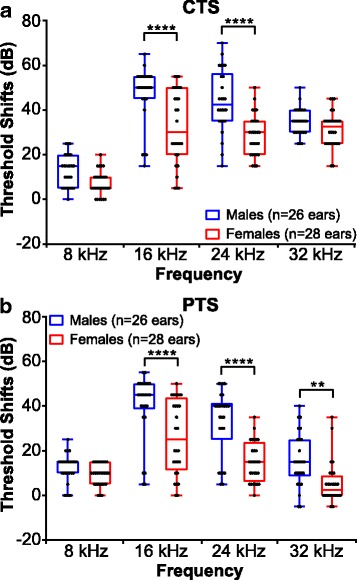
Male and female B6CBAF1/J mice respond differently to 101 dB SPL octave band noise. Threshold shifts were compared between vehicle-treated male and female mice. The dots indicate individual ear threshold shifts, the upper and lower whiskers indicate the maximum and minimum shifts, respectively. **a** CTS were significantly lower in females when compared to males at 16 and 24 kHz (****p < 0.0001). **b** PTS values were significantly reduced in females at 16, 24 (****p < 0.001), and 32 kHz (**p < 0.01)

To test whether male and female mice have a differential response to noise, we first compared baselines in males and females to rule out a possible difference in hearing thresholds before noise exposure (Fig. 2a and Additional file 2: Table S1). A two-way ANOVA followed by a post hoc analysis detected a small but significant lower threshold in females at 32 kHz (*p* = 0.0008; *d* = 0.357) but not at 8, 16, and 24 kHz. We next assessed the CTS and PTS for each sex separately (Fig. 2b and Additional file 2: Table S2). Similar to the results obtained with both sexes combined, males and females had significant CTS and PTS at all frequencies measured when compared to pre-noise baseline. However, when male and female threshold shifts following noise exposure were compared to each other, a two-way ANOVA revealed main effects of frequency and sex on both CTS (frequency: *F*_3, 208_ = 84.84; *p* < 0.0001; sex: *F*_1, 208_ = 43.41; *p* < 0.0001) and PTS (frequency: *F*_3, 208_ = 47.46; *p* < 0.0001; sex: *F*_1, 208_ = 49.58; *p* < 0.0001) (Fig. 3 and Additional file 2: Table S3). A *post hoc* comparison revealed that 24 h after noise exposure, females have a significantly lower CTS at 16 and 24 kHz (*p* < 0.0001 for both frequencies; *d* = 0.676 for 16 kHz and *d* = 0.727 for 24 kHz) compared to males (Fig. 3 and Additional file 2: Table S4). This difference is extended to the 32 kHz frequency as well 15 days post-noise exposure (*p* < 0.0001 for 16 and 24 kHz; *p* = 0.005 for 32 kHz; *d* = 0.598 for 16 kHz, *d* = 0.779 for 24 kHz, and *d* = 0.453 for 32 kHz). These data suggest that females have a less severe hearing loss following noise exposure compared to males.

### Effect of noise on ABR wave I amplitude in male and female mice

**Fig. 4:**
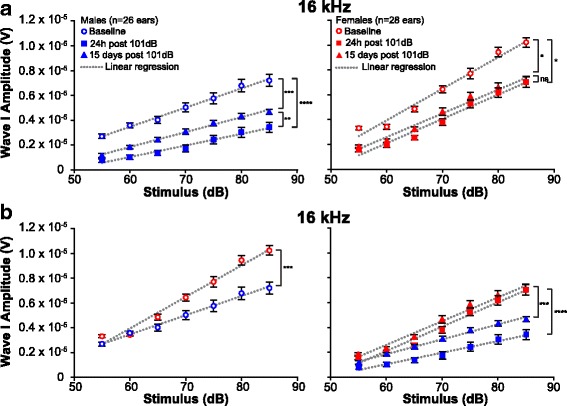
Differences in the slopes of ABR wave I amplitudes between male and female mice at 16 kHz. Growth of ABR wave I amplitude as a function of increasing stimuli levels at 16 kHz was compared by analysis of the slopes from linear regression of the data (dotted lines). The slopes are shown at baseline, 24 h, and 15 days post-noise exposure in males and females. **a** Slopes are reduced following noise exposure when compared to baseline for males (left) and females (right). Males partially recover at 15 days when compared to 24 h while females do not. **b** At baseline (left), the slope value from female wave I amplitude is higher than the value from males. The difference is maintained following noise exposure (right) at 24 h and 15 days. *p < 0.05; **p < 0.01; ***p < 0.001; ****p < 0.0001. Error bars indicate S.E.M. ns, non-significant

The cochlea has two types of sensory cells—inner hair cells (IHC) and outer hair cells (OHC). The OHC function primarily as signal amplifiers, whereas the IHC receive primarily afferent innervation and are the main source of auditory sensory input to the brain [41]. The ABR wave I amplitude is primarily a reflection of the frequency-specific activity at the spiral ganglion (SG), which is the ganglion that houses the cell bodies of the afferent neurons that come in contact mainly with the IHC. This activity is a compound of the levels of IHC and OHC activity (as IHC activity is influenced by OHC function), the number of active auditory nerve fibers present, functional synapses, as well as the endocochlear potential [17, 42]. To assess whether the difference in the male and female response to noise correlates with a difference in the synchronous activity at the SG, the increase in ABR wave I amplitude as a function of increasing sound level was measured in the vehicle-treated noise-exposed mice. A change in amplitude can result from changes in any of the factors that contribute to wave I. We analyzed wave I amplitude at the frequency with the maximal threshold shift, which was 16 kHz in this study. Average peak-to-trough wave I amplitudes were extracted for stimuli levels 55 dB to 85 dB for each animal at baseline (prior to noise exposure), 24 h, and 15 days post-noise exposure. A change in the slope of the amplitude as a function of sound level would most likely reflect a change in active processes in the cochlea, primarily attributed to OHC function [43, 44]. (Fig. 4). A linear regression analysis showed a decrease in the slope 24 h post-noise exposure for both males and females. At baseline, males had an average slope of 155 ± 6 nV/dB, while at 24 h post-noise exposure, this slope significantly decreased to 93 ± 6 nV/dB (*p* < 0.0001) (Fig. 4a). The average slope at baseline for females was 255 ± 16 nV/dB as compared to 196 ± 14 nV/dB for 24 h post-noise exposure (*p* = 0.0022) (Fig. 4a). At 15 days post-noise exposure, the slope value partially recovered in males when compared to 24 h, averaging 120 ± 4 nV/dB (*p* = 0.0051), but remained significantly lower than baseline (*p* = 0.0008) (Fig. 4a). While males recovered partially, the average slope for females at 15 days post-noise exposure was similar to the slope at 24 h with a value of 191 ± 13 nV/dB (*p* = 0.7959). Interestingly, when we directly compared the slopes between males and females from the same time point, a two-tailed *t* test revealed significant differences between the slopes (Fig. 4b). At baseline, females have a slope of 255 ± 16 nV/dB as compared to a slope of 155 ± 6 nV/dB for males (*p* = 0.0002). This difference is maintained after noise exposure at 24 h and 15 days (Fig. 4b). Comparison of the absolute amplitude of wave I at 85 dB SPL shows permanent lower amplitude in the males compared with the female mice (Fig. 4b and Table 1).

**Table 1:** Values for the wave I absolute amplitudes at 85 dB SPL

	Wave I amplitudes at a 85 dB stimulus (volt)		
	Baseline	24 h post 101 dB	15 days post 101 dB
Males	7.21 × 10^−6^	3.42 × 10^−6^	4.67 × 10^−6^
Females	10.2 × 10^−6^	7.04 × 10^−6^	7.04 × 10^−6^
*p* value	< 0.0001	< 0.0001	< 0.0001
*d* value	1.380	1.719	1.317

Females have a higher amplitude at baseline, 24 h, and 15 days post-noise exposure (unpaired *t* test)

### Effect of noise on OHC loss

**Fig. 5:**
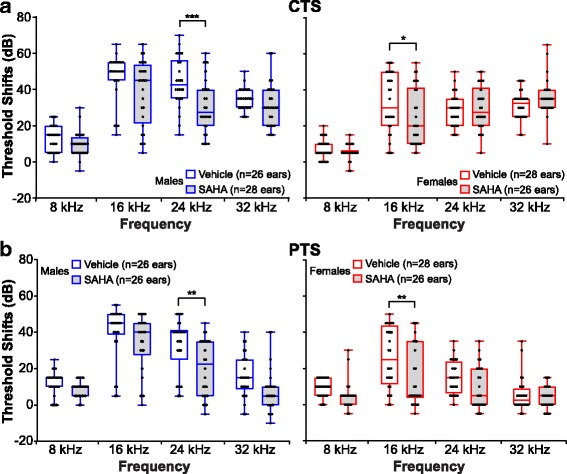
Protective effect of SAHA on NIHL—separated by sex. Threshold shifts were compared between SAHA- and vehicle-treated males (left) and females (right) at 24 h (**a**) and 15 days (**b**) post-noise exposure. The dots indicate individual ear threshold shifts, the upper and lower whiskers indicate the maximum and minimum shifts, respectively. **a** CTS values for males (left) and females (right). Male mice are protected only at 24 kHz (***p < 0.001) and female mice only at 16 kHz (*p < 0.05). **b** PTS values for males (left) and females (right). Females remain protected at 16 kHz (** *p* < 0.01) and males remain protected at 24 kHz (**p < 0.01)

To identify possible causes for the differential response to noise between male and female mice, we performed cytocochleograms to compare hair cell loss throughout the cochlear duct (up to a frequency position corresponding to 55 kHz) (Additional file 3). For unexposed controls, we used strain (B6CBAF1/J) and age-matched (12 weeks old) mice. As expected, 12-week-old control mice (males and females) showed little to no OHC loss along the organ of Corti (0 to 0.34% loss) (Table 2 and Additional file 4). Similarly, 2 weeks following a 101 dB noise exposure, there was no significant OHC loss (less than 1%) in either sex up to 32 kHz (Fig. 5). While an OHC loss was measured at 32–55 kHz (Table 2 and Additional file 4), no sex-specific differences were measured with respect to OHC loss (Table 2, Additional file 4 and Additional file 2: Table S5). These results suggest that the sex difference seen in response to noise exposure is not explained by a divergence in OHC loss in males and females. Interestingly, the pattern of OHC loss seen in noise-exposed animals does not match the frequency-specific PTS. The cochlea is organized such that high-frequency sounds are sensed at the base of the organ, close to the “entry of sound,” and low-frequency sounds at the apex (also known as a tonotopic organization) [45]. While the highest PTS is measured at 16 and 24 kHz, only minimal OHC loss is observed around these frequencies (Table 2 and Additional file 4). Therefore, our data indicate that the OHC loss at the 16 and the 24 kHz location is not sufficient to explain the PTS at these frequencies when measured 15 days post-noise exposure.

**Table 2:** Values for the percentage of OHC loss within nine frequency ranges measured

		OHC loss by frequency range								
		4–5.6 kHz	5.6-8 kHz	8–11.3 kHz	11.3-16 kHz	16–22.6 kHz	22.6-32 kHz	32–45.2 kHz	45.2-51 kHz	51-55 kHz
Controls	Males	0.12 ± 0.12	0.23 ± 0.23	0.34 ± 0.21	0.03 ± 0.03	0.26 ± 0.16	0.23 ± 0.08	0.29 ± 0.10	0.34 ± 0.34	0.15 ± 0.15
	Females	0.00 ± 0.00	0.13 ± 0.09	0.06 ± 0.06	0.09 ± 0.06	0.07 ± 0.05	0.10 ± 0.07	0.18 ± 0.12	0.00 ± 0.00	0.18 ± 0.18
DMSO + noise	Males	0.29 ± 0.20	0.23 ± 0.12	0.16 ± 0.08	0.15 ± 0.05	0.22 ± 0.07	0.77 ± 0.23	4.76 ± 1.17	21.8 ± 4.57	45.7 ± 10.3
	Females	0.00 ± 0.00	0.02 ± 0.02	0.20 ± 0.08	0.10 ± 0.06	0.35 ± 0.11	0.43 ± 0.16	5.36 ± 2.32	22.4 ± 7.73	45.3 ± 14.2

Progressive OHC loss is seen beginning from 32 kHz. Both male and female animals show a similar pattern of OHC loss. ± represent S.E.M

### Effect of noise trauma on IHC synapses

Our data indicate that 2 weeks after noise exposure, at a time a PTS is already obtained, the OHC loss does not account for either the significant PTS at 16 and 24 kHz or the sex differences observed in the PTS and wave I amplitude progression. Loss of IHC functional synapses has been shown to account for the decrease in wave I amplitude following lower intensity noise exposures, a phenomenon also known as cochlear synaptopathy [46]. We therefore focused our analysis on the IHC synapses. We first quantified the number of pre-synaptic ribbons in the IHCs. For this purpose, whole mount cochleae were fluorescently immunolabeled with an antibody directed against CtBP2 to visualize the pre-synaptic ribbons. Pre-synaptic ribbons were counted on Z-stacks created from confocal sections in the regions of 16, 24, and 32 kHz (Additional file 5). In the region where the maximal threshold shift is detected (16 kHz location), no significant change in the pre-synaptic ribbons was recorded following noise in either sex (Table 3 and Additional file 6). However, a significant decrease in the pre-synaptic ribbons per IHC was observed in the region of 24 and 32 kHz in both male and female mice (Table 3 and Additional file 6). Interestingly, no difference was detected between males and females at the three frequencies analyzed in either the controls or noise-exposed animals (*p* > 0.05), suggesting that the change in pre-synaptic ribbons does not account for the sex differences in hearing following noise exposure.

**Table 3:** Pre-synaptic ribbon counts per IHC in the 16, 24 and 32 kHz regions in male and female mice

	Total IHC ribbons								
	16 kHz location			24 kHz location			32 kHz location		
	Males	Female	*p* value	Males	Females	*p* value	Males	Females	*p* value
Controls	15.00 ± 0.72	15.86 ± 0.53	0.34	16.16 ± 0.78	15.85 ± 0.61	0.76	15.60 ± 0.98	13.78 ± 0.92	0.20
DMSO + Noise	15.25 ± 0.86	14.60 ± 0.87	0.60	11.38 ± 0.74	11.95 ± 0.95	0.64	7.65 ± 0.88	8.11 ± 0.78	0.71

± represent S.E.M. (unpaired *t* test to compare males versus females)

Recent evidence suggests that noise exposure reduces the number of active IHC synapses by inducing the retraction of some of the neurons that come in contact with the IHC. Shortly after noise exposure, while the synaptic ribbons may persist, loss of neuronal contact can be identified by loss of post-synaptic densities [47]. We therefore analyzed the number of pre-synaptic ribbons paired with post-synaptic glutamate receptor (GluR2) to determine if the different response to noise exposure between male and female mice can be attributed to the number of active synapses (Additional file 5). Similar to pre-synaptic ribbons, noise exposure induced a significant decrease in the number of active synapses at the regions of 24 and 32 kHz but not 16 kHz. However, again, there was no sexual dimorphism in the number of active synapses (Table 4 and Additional file 6). These data indicate that the noise-induced synaptopathy is not the main underlying cause for the PTS seen at 16 kHz, which is the frequency with the maximal threshold shift, and is not the culprit of the sexual dimorphism in the response to noise.

**Table 4:** Active synapse counts per IHC at 16, 24, and 32 kHz in male and female mice

	Active synapses								
	16 kHz location			24 kHz location			32 kHz location		
	Males	Females	*p* value	Males	Females	*p* value	Males	Females	*p* value
Control	13.26 ± 1.75	14.51 ± 2.82	0.63	14.84 ± 1.91	13.64 ± 1.11	0.60	11.36 ± 1.38	9.73 ± 1.07	0.41
DMSO + Noise	11.66 ± 1.38	11.39 ± 1.50	0.89	8.47 ± 1.28	7.34 ± 2.13	0.66	3.94 ± 1.18	4.86 ± 0.99	0.58

± represent S.E.M. (unpaired *t* test to compare males versus females)

### Sex influences the measured effect of SAHA treatment on mice exposed to noise

To date, most studies on noise exposure and its treatments were performed on male mice only or mice of both sexes combined. Because our data show a differential response to noise between male and female mice, we next explored whether sex influences the measured response to treatment. This is important for proper testing of therapeutics. To determine whether SAHA has a protective effect from noise exposure, CTS (Fig. 5a) and PTS values (Fig. 5b) were compared between vehicle and SAHA-treated animals. A two-way ANOVA revealed main effects of SAHA and sex on CTS at 8, 16, and 24 kHz (Table 5 and Additional file 2: Table S3). Main effects of SAHA and sex 15 days post-noise exposure is significant at all frequencies tested (Table 5).

**Table 5:** Main effects of SAHA and sex on CTS and PTS following a two-way ANOVA

		8 kHz		16 kHz		24 kHz		32 kHz	
		F	*p* value	F	*p* value	F	*p* value	F	*p* value
CTS	Sex	18.67	**< 0.0001**	20.06	**< 0.0001**	11.07	**0.001**	0.174	ns
	SAHA	4.845	**0.030**	6.728	**0.011**	5.678	**0.020**	0.102	ns
PTS	Sex	6.885	**0.010**	26.35	**< 0.0001**	22.14	**< 0.0001**	14.02	**0.0003**
	SAHA	9.089	**0.003**	6.723	**0.011**	13.18	**0.0004**	5.174	**0.025**

For CTS and PTS, the degree of freedom for the numerator is 1. The degrees of freedom for the denominator are 104 and 102 for CTS and PTS, respectively. Significant results are shown in bold font (*ns* non-significant)

Post hoc comparisons revealed that CTS of SAHA-treated males were significantly lower at 24 kHz (*p* = 0.0006; *d* = 0.536) compared to vehicle-treated controls, whereas in females, CTS values were significantly lower at 16 kHz (*p* = 0.04; *d* = 0.359) (Fig. 5a**,** Additional file 2: Table S6). Comparisons of PTS suggested that the protective effect of SAHA in male mice is maintained at 24 kHz (*p* = 0.002; *d* = 0.489) and at 16 kHz in female mice (*p* = 0.003; *d* = 0.482) compared to the vehicle-treated controls (Fig. 5b, Additional file 2: Table S6). These data indicate a difference in the response to SAHA between male and female mice.

**Fig. 6:**
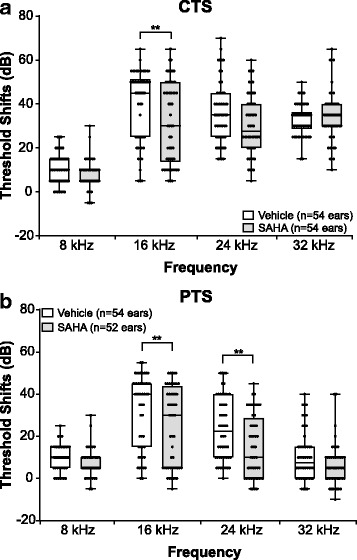
Protective effect of SAHA on NIHL—both sexes combined. Threshold shifts were compared between SAHA- and vehicle-treated animals at 24 h (**a**) and 15 days (**b**) post-noise exposure. The dots indicate individual ear threshold shifts; the upper and lower whiskers indicate the maximum and minimum shifts, respectively. **a** CTS values suggested a protective effect of SAHA at 16 kHz (*p = 0.007). **b** PTS values suggested a protective effect of SAHA at 16 kHz (*p = 0.0095) and 24 kHz (**p = 0.0024)

Next, we re-analyzed the data, this time combining mice from both sexes, to assess whether this might change the measured response to treatment. A two-way ANOVA revealed significant main effects of SAHA and frequency at 24 h (SAHA: *F*_1, 424_ = 9.576, *p* = 0.0021; frequency: *F*_3, 424_ = 110.1, *p* < 0.0001) and 15 days (SAHA: *F*_1, 416_ = 22.67, *p* < 0.0001; frequency: *F*_3, 416_ = 57.36, *p* < 0.0001) post-noise exposure. Compared to vehicle-treated controls, SAHA significantly decreased the CTS only at 16 kHz (*p* = 0.0074) (Fig. 6a, Additional file 2: Table S7) while a significant decrease in PTS was observed at 16 and 24 kHz (*p* = 0.0095 and 0.0024, respectively) (Fig. 6b, Additional file 2: Table S7). Thus, these findings indicate that when combining mice from both sexes, the measured response to treatment is different from the response when each sex is analyzed separately. This is critically important as it may lead to misinterpretation of biological data.

## Discussion

The major finding reported here is the identification and characterization of a sexually dimorphic response to PTS-inducing noise exposure and its candidate therapeutics in mice. Sex is an important biological variable with effects on a wide range of physiological processes, and it must be considered in the experimental design to allow study results to relate to both male and female biology [48]. Importantly, the National Institutes of Health has added consideration of sex as a biological factor in all applications considered for funding [49, 50]. We tested the efficacy of SAHA on prevention of noise-induced hearing loss in mice of both sexes. SAHA has been previously shown to be otoprotective against ototoxic drugs [51–54] and NIHL in male mice [14, 31]. Our results confirmed the efficacy of SAHA in male mice, albeit possibly to a lesser degree than previously reported [14, 31], and revealed only a small protective effect in females. Importantly, our treatment paradigm differed from previously published work and could account for some of the difference in efficacy. When the PTS from both sexes were analyzed together, 15 days post-noise exposure, a statistically significant protective effect of SAHA was found at both 16 and 24 kHz. However, when the data were separated by sex, we found that the protective effect of SAHA in males was limited to 24 kHz while in females to 16 kHz. Female mice demonstrated less hearing loss in response to noise at 16 and 24 kHz, in comparison to males, suggesting a sex-specific difference in the response to PTS-inducing noise trauma. This sex difference may explain the differential frequency-specific therapeutic efficacy of SAHA, where males at 16 kHz may have suffered too much damage to allow for SAHA-dependent rescue, and females at 24 kHz have too little PTS to allow a therapeutic effect to be detected with the number of mice tested. Concordantly, previous studies suggested a level-specific limitation to the therapeutic effect of SAHA [14, 31].

To further investigate the sex-specific differences in hearing following PTS-inducing noise exposure, we compared OHC loss, wave I amplitude, and amplitude progression, as well as IHC pre-synaptic ribbons and active synapses. To our surprise, we found a significant difference between the sexes only in the wave I amplitude and amplitude progression. Wave I amplitude is an indicator of activity at the level of the SG, whereas wave I amplitude progression reflects the OHC contribution to the active process of hearing. Since the number of hair cells and synapses following noise exposure was not different across sexes, a decrease in wave I amplitude and amplitude progression suggests a greater decrease in OHC activity in the male mice. The suggested decrease in OHC function may be primary and represent a dysfunction resulting from injury to the stereocilia or cell bodies [46], or secondary as a consequence of changes in the endocochlear potential. A recent study in F344 rats shows that the difference in hearing loss between aging male and female animals results in part from cellular degeneration at the level of the stria vascularis [55]. However, in this strain of rats OHC loss progressed from apex to base, indicating that the pathophysiology underlying the ARHL in the F344 rats may not be generalizable. Additional studies using inbred mouse strains revealed a divergent pathophysiology for male and female age-related loss [56, 57]. These observations suggest that outbred mice such as the B6CBAF1/J may prove particularly useful in the study of NIHL, as the effect of strain-specific recessively inherited mutations on the auditory system will be largely avoided. Future studies comparing changes in OHC and stria vascularis morphology and ultrastructure, as well as measurement of the endocochlear potential and distortion product otoacoustic emissions (DPOAE), are necessary to further define the underlying sex-specific differences following noise exposure [58, 59].

**Fig. 7:**
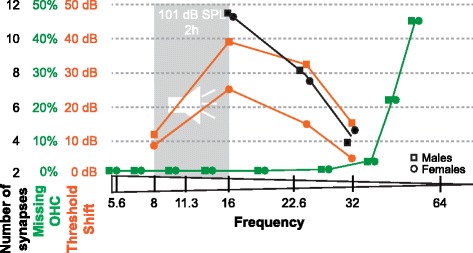
Schematic showing the threshold shifts in relation to missing outer hair cells and number of active synapses. Loss of OHC (green) and decrease in the number of active synapses (black) does not correlate with the highest threshold shift (orange) following noise exposure detected at 16 kHz. The difference in threshold shifts between male and female mice is not explained by a difference in OHC loss or active synapse numbers

We employed an octave band (8–16 kHz) noise exposure paradigm that results in PTS in the B6CBAF1/J mouse strain. As expected following these type of exposures, we measured a maximal threshold shift at 16 and 24 kHz. A smaller threshold shift was measured at 32 kHz, the highest frequency analyzed in this study. A marked and significant loss of OHC was seen in both sexes in regions that correspond to frequencies higher than 32 kHz; however, only a minimal loss of OHC was measured in areas that map to 16 and 24 kHz. Taken together, these data suggest that two weeks following noise exposure, there are two types of hearing loss that differ in their underlying mechanisms. A loss of OHC in the base of the cochlea underlies a high-frequency hearing loss, which is not directly related to the frequency of the noise exposure. Rather, it represents a non-specific acoustic injury likely secondary to the position and physical characteristics of the cells in the base of the cochlea. In addition, a frequency-specific PTS, which is the focus of this manuscript, is not secondary to loss of OHC. More importantly, these findings suggest a possible therapeutic window to treat the OHC and possibly prevent the frequency-specific PTS, as OHC in the frequency-specific PTS are still present 2 weeks following exposure. Later degeneration of OHC following PTS has been observed when ears are analyzed 1 year following noise exposure [18]. Similar to the lack of OHC loss at 16 kHz, the frequency where maximal PTS is found in both sexes, we did not observe a significant loss of active synapses in either sex. A progressive loss of active synapses was seen at 24 and 32 kHz. These data suggest that at least 2 weeks following PTS-inducing noise exposure, synaptopathy preferentially affects higher frequencies and does not explain the loss of hearing at 16 kHz or the sex-specific differences in the response to noise trauma (Fig. 7).

Differences in circulating levels of the steroid hormone estradiol and/or sensitivity to estradiol via its receptor activation may account for the observed sex difference in PTS as a result of noise trauma. Evidence from both clinical and basic studies clearly demonstrates that estradiol plays an important role in modulating auditory function in vertebrates as well as conferring a protective function in the female auditory system [60]. Estradiol signaling primarily occurs via two cognate receptors that are ligand-activated transcription factors. Estrogen receptor 1 (ERS1) and estrogen receptor 2 (ERS2) are widely distributed throughout the body and both have been reported in the cochlea of rodents and humans [61–64]. In mice, ESR1 and ESR2 are present in both the inner and outer hair cells, as well as the spiral ganglion neurons [61, 63, 65]. ESR2 and not ESR1 has been implicated in conferring the protective actions of estradiol against temporary hearing loss as result of a noise trauma in male and female mice [65] and age-related hearing loss in female mice [66]. However, the exact mechanisms through which estradiol is acting to confer protection is not well understood. In addition, molecular differences independent of the estradiol signaling pathway should also be considered in the underpinning mechanisms of the sexual dimorphic response to noise exposure.

## Conclusions

In conclusion, this study documents significant sex-specific differences in the response of the mouse cochlea to damaging noise exposure. These findings have implications on future study design for proper interpretation of the data. In particular, male and female mice should be tested and analyzed separately when used to study NIHL. As females demonstrate less noise-induced hearing damage in comparison to males, they may require exposure to a higher sound level to assess therapeutic effects. In addition, understanding the underpinnings of the females’ relative protection from NIHL could lead to the development of new therapeutics to ameliorate the outcomes of noise exposures. Classic approaches to interrogate sex differences such as studying gonadectomized mice with and without supplemental sex hormones might be particularly useful to overcome challenges related to fluctuation of circulating sex hormones [67].

## Additional files

Additional file 1: Schematic showing ABR wave I extraction and analysis. Peak (P1) and trough (T1) values of wave I of the ABR traces (wave shaded in blue) were automatically extracted at stimuli levels from 55 to 85 dB SPL using a MatLab script. Wave I amplitudes were then plotted as a function of the stimuli levels. SigmaPlot was used to perform linear regression (dotted line) and calculate the slope (solid lines). Slopes were then compared between the different groups at 16 kHz. (PDF 471 kb)

Additional file 2: **Table S1.**Comparison of average hearing thresholds at baseline in male and female mice (Sidak’s multiple comparison test; ns: non-significant). **Table S2.** Average threshold shift values in dB at 24 h post-noise exposure (CTS) and 15 days post-noise exposure (PTS) in vehicle-treated males and females (Tukey’s multiple comparisons test). **Table S3.** Statistical values for interactions between the two factors following a two-way ANOVA. The degrees of freedom for the numerator (DFn) and denominator (DFd) are shown in parenthesis before the *F* value. Significant results are shown in bold font. **Table S4.** Comparison of average ABR thresholds shifts at 24 h (CTS) and 15 days (PTS) post-noise exposure in male and female mice treated with vehicle only (Sidak’s multiple comparison test; ns: non-significant). **Table S5.** Values for the percentage of OHC loss within 32–45.2 kHz, 45.2–51 kHz, and 51–55 kHz. Progressive OHC loss is seen up to 55 kHz which is the highest frequency counted. Both male and female animals show a similar pattern of OHC loss. ± represent S.E.M. (unpaired *t* test to compare male and female mice). **Table S6.** Comparison of average threshold shift values in dB at 24 h post-noise exposure (CTS) and 15 days post-noise exposure (PTS) between vehicle- and SAHA-treated males and females separately (Sidak’s multiple comparisons test; ns: non-significant). **Table S7.** Comparison of average threshold shift values in dB at 24 h post-noise exposure (CTS) and 15 days post-noise exposure (PTS) between vehicle- and SAHA-treated animals (Sidak’s multiple comparisons test; ns: non-significant). (PDF 257 kb)

Additional file 3: OHC loss along the cochlear duct. Representative fluorescence microscopy images of the Organ of Corti at the level of the OHC (counter-stained with DAPI) at different frequency bands from controls and mice exposed at 101 dB SPL. There is little to no OHC loss in the control animals, whereas extensive OHC loss is seen above 32 kHz in animals exposed to noise. Scale bar represents 20 μm. (PDF 1120 kb)

Additional file 4: OHC loss does not account for the frequency-specific PTS at 16 and 24 kHz or the sex differences in NIHL. Line graph indicating the percentage of OHC loss from apex to base in vehicle-treated noise-exposed animals compared to control non-noise-exposed animals. The frequency range of noise exposure is shaded gray and a gray dotted line outlines the frequency range where significant PTS is seen. Error bars indicate S.E.M. (PDF 396 kb)

Additional file 5: Pre-synaptic ribbons and active synapses at 16 kHz and 24 kHz. Representative fluorescence microscopy images of IHC stained for CtBP2 (red) and GluR2 (green) at 16 kHz (left) and 24 kHz (right) from control and noise-exposed mice. The dotted lines represent the approximate border of one IHC. The inset in the bottom left corner image represent a zoom in of active synapses where CtBP2 and GluR2 partially co-localize. Scale bar represents 10 μm. (PDF 1242 kb)

Additional file 6: Effect of noise on pre-synaptic ribbons and active synapses in IHC. Graphs representing the number of pre-synaptic ribbons **(a)** and active synapses **(b)** in IHC of control and vehicle-treated noise-exposed animals. A significant decrease in pre-synaptic ribbons and active synapses is observed at 24 and 32 kHz in both males and females, but no difference is seen between sexes. Error bars indicate S.E.M. (PDF 418 kb)

## Abbreviations

ABR: Auditory brainstem response
ARHL: Age-related hearing loss
CTS: Compound threshold shift
dB: Decibel
DMSO: Dimethyl sulfoxide
DPOAE: Distortion product otoacoustic emissions
IHC: Inner hair cell
NILH: Noise-induced hearing loss
OHC: Outer hair cell
PTS: Permanent threshold shift
SAHA: Suberoylanilide hydroxamic acid
SPL: Sound pressure level

## References

[1] Nelson DI, Nelson RY, Concha-Barrientos M, Fingerhut M (2005). The global burden of occupational noise-induced hearing loss. Am J Ind Med.

[2] Carroll YI, Eichwald J, Scinicariello F, Hoffman HJ, Deitchman S, Radke MS (2017). Vital signs: noise-induced hearing loss among adults—United States 2011–2012. MMWR Morb Mortal Wkly Rep.

[3] Lie A, Skogstad M, Johannessen HA, Tynes T, Mehlum IS, Nordby K-C (2016). Occupational noise exposure and hearing: a systematic review. Int Arch Occup Environ Health.

[4] U.S. Department of Veterans Affairs (2016). Veterans benefit administration, annual benefits report, fiscal year 2016.

[5] Lin FR, Hazzard WR, Blazer DG (2016). Priorities for improving hearing health care for adults: a report from the National Academies of Sciences, Engineering, and Medicine. JAMA J Am Med Assoc.

[6] Salvi R, Boettcher FA, Conn PM (2008). Animal models of noise-induced hearing loss. Sourceb. Model. Biomed. Res.

[7] Nodal FR, King AJ. Hearing and auditory function in ferrets. In: Fox JG, Marini R, editors. Biol Dis Ferret. 3rd ed. Ames: Wiley; 2014. p. 685–710.

[8] Depireux DA, Simon JZ, Klein DJ, Shamma SA (2001). Spectro-temporal response field characterization with dynamic ripples in ferret primary auditory cortex. J Neurophysiol.

[9] Chen G-D, Fechter LD (2003). The relationship between noise-induced hearing loss and hair cell loss in rats. Hear Res.

[10] Ohlemiller KK (2006). Contributions of mouse models to understanding of age- and noise-related hearing loss. Brain Res.

[11] Ahituv N, Avraham KB (2000). Auditory and vestibular mouse mutants: models for human deafness. J Basic Clin Physiol Pharmacol.

[12] Layman WS, Williams DM, Dearman JA, Sauceda MA, Zuo J (2015). Histone deacetylase inhibition protects hearing against acute ototoxicity by activating the Nf-κB pathway. Cell Death Discov.

[13] Xu WS, Parmigiani RB, Marks PA (2007). Histone deacetylase inhibitors: molecular mechanisms of action. Oncogene.

[14] Chen J, Hill K, Sha S-H (2016). Inhibitors of histone deacetylases attenuate noise-induced hearing loss. JARO.

[15] Yoshida N, Kristiansen A, Liberman MC (1999). Heat stress and protection from permanent acoustic injury in mice. J Neurosci.

[16] Tahera Y, Meltser I, Johansson P, Canlon B (2006). Restraint stress modulates glucocorticoid receptors and nuclear factor kappa B in the cochlea. Neuroreport.

[17] Kujawa S, Liberman C (2009). Adding insult to injury: cochlear nerve degeneration after “temporary” noise-induced hearing loss. J Neurosci.

[18] Fernandez KA, Jeffers PWC, Lall K, Liberman MC, Kujawa SG (2015). Aging after noise exposure: acceleration of cochlear synaptopathy in “recovered” ears. J Neurosci.

[19] Lauer AM, Schrode KM (2017). Sex bias in basic and preclinical noise-induced hearing loss research. Noise Health.

[20] Canlon B, Meltser I, Johansson P, Tahera Y (2007). Glucocorticoid receptors modulate auditory sensitivity to acoustic trauma. Hear Res.

[21] Jerger J, Chmiel R, Stach B, Spretnjak M (1993). Gender affects audiometric shape in presbyacusis. J Am Acad Audiol.

[22] Ward WD, Royster JD, Royster LH. Auditory and nonauditory effects of noise. In: Berger EH, Royster LH, Royster JD, Driscoll DP, Layne M, editors. Noise Man. 5th ed: Fairfax: American Industrial Hygiene Association; 2000. p. 101–47.

[23] Waterston RH, Lindblad-Toh K, Birney E, Rogers J, Abril JF, Agarwal P (2002). Initial sequencing and comparative analysis of the mouse genome. Nature.

[24] Brown SDM, Hardisty-Hughes RE, Mburu P (2008). Quiet as a mouse: dissecting the molecular and genetic basis of hearing. Nat Rev Genet.

[25] Scimemi P, Santarelli R, Selmo A, Mammano F (2014). Auditory brainstem responses to clicks and tone bursts in C57 BL/6J mice. Acta Otorhinolaryngol Ital.

[26] Noben-Trauth K, Zheng QY, Johnson KR (2003). Association of cadherin 23 with polygenic inheritance and genetic modification of sensorineural hearing loss. Nat Genet.

[27] Davis RR, Newlander JK, Ling XB, Cortopassi GA, Krieg EF, Erway LC (2001). Genetic basis for susceptibility to noise-induced hearing loss in mice. Hear Res.

[28] Johnson KR, Zheng QY, Noben-Trauth K (2006). Strain background effects and genetic modifiers of hearing in mice. Brain Res.

[29] Ou HC, Bohne BA, Harding GW (2000). Noise damage in the C57BL/CBA mouse cochlea. Hear Res.

[30] Longenecker RJ, Galazyuk AV (2012). Methodological optimization of tinnitus assessment using prepulse inhibition of the acoustic startle reflex. Brain Res.

[31] Wen L-T, Wang J, Wang Y, Chen F-Q (2015). Association between histone deacetylases and the loss of cochlear hair cells: role of the former in noise-induced hearing loss. Int. J. Mol. Med.

[32] Ponton CW, Moore JK, Eggermont JJ (1996). Auditory brain stem response generation by parallel pathways: differential maturation of axonal conduction time and synaptic transmission. Ear Hear.

[33] Zuccotti A, Lee SC, Campanelli D, Singer W, Satheesh SV, Patriarchi T (2013). L-type CaV1.2 deletion in the cochlea but not in the brainstem reduces noise vulnerability: implication for CaV1.2-mediated control of cochlear BDNF expression. Front Mol Neurosci.

[34] Fuchs JC, Zinnamon FA, Taylor RR, Ivins S, Scambler PJ, Forge A, et al. Hearing loss in a mouse model of 22q11.2 deletion syndrome. PLoS One. 2013;8:e80104. http://journals.plos.org/plosone/article?id=10.1371/journal.pone.0080104. 10.1371/journal.pone.0080104 PMC3828191 24244619

[35] Ohlemiller KK, Jones SM, Johnson KR (2016). Application of mouse models to research in hearing and balance. JARO - J. Assoc. Res. Otolaryngol.

[36] Zar JH (1984). Biostatistical analysis.

[37] Massachusetts Eye and Ear (2015). Video tutorial for mouse cochlear dissection.

[38] Massachusetts Eye and Ear. ImageJ Plugin Measure_Line for mapping cochlear frequencies on whole mounts. https://www.masseyeandear.org/research/otolaryngology/investigators/laboratories/eaton-peabody-laboratories/epl-histology-resources/imagej-plugin-for-cochlear-frequency-mapping-in-whole-mounts.

[39] Abràmoff MD, Magalhães PJ, Ram SJ (2004). Image processing with imageJ. Biophotonics Int.

[40] Abdi H. The Bonferroni and Šidák corrections for multiple comparisons. In: Salkind N, editor. Encycl Meas Stat. Thousand Oaks: Sage Publications; 2007. p. 103–7. https://www.utd.edu/~herve/Abdi-Bonferroni2007-pretty.pdf.

[41] Goutman JD, Elgoyhen AB, Gómez-Casati ME (2015). Cochlear hair cells: the sound-sensing machines. FEBS Lett.

[42] Henry KS, Kale S, Scheidt RE, Heinz MG. Auditory brainstem responses predict auditory nerve fiber thresholds and frequency selectivity in hearing impaired chinchillas. Hear Res. 2011;280:236–44. 10.1016/j.heares.2011.06.002 PMC3179834 21699970

[43] Hudspeth A (1997). Mechanical amplification of stimuli by hair cells. Curr Opin Neurobiol.

[44] Martin P, Hudspeth AJ (1999). Active hair-bundle movements can amplify a hair cell’s response to oscillatory mechanical stimuli. Proc Natl Acad Sci U S A.

[45] Mann ZF, Kelley MW (2011). Development of tonotopy in the auditory periphery. Hear Res.

[46] Kujawa SG, Liberman MC (2015). Synaptopathy in the noise-exposed and aging cochlea: primary neural degeneration in acquired sensorineural hearing loss. Hear Res.

[47] Suzuki J, Corfas G, Liberman MC. Round-window delivery of neurotrophin 3 regenerates cochlear synapses after acoustic overexposure. Sci Rep. 2016;6:24907. http://www.nature.com/articles/srep24907. 10.1038/srep24907 PMC4842978 27108594

[48] Karp NA, Mason J, Beaudet AL, Benjamini Y, Bower L, Braun RE (2017). Prevalence of sexual dimorphism in mammalian phenotypic traits. Nat Commun.

[49] Hultcrantz M, Simonoska R, Stenberg AE (2006). Estrogen and hearing: a summary of recent investigations. Acta Otolaryngol.

[50] Clayton JA, Collins FS (2014). Policy: NIH to balance sex in cell and animal studies. Nature.

[51] Drottar M, Liberman MC, Ratan RR, Roberson DW (2006). The histone deacetylase inhibitor sodium butyrate protects against cisplatin-induced hearing loss in guinea pigs. Laryngoscope.

[52] Chen FQ, Schacht J, Sha SH (2009). Aminoglycoside-induced histone deacetylation and hair cell death in the mouse cochlea. J Neurochem.

[53] Wang J, Wang Y, Chen X, Zhang PZ, Shi ZT, Wen LT (2015). Histone deacetylase inhibitor sodium butyrate attenuates gentamicin-induced hearing loss in vivo. Am J Otolaryngol Head Neck Med Surg.

[54] Layman WS, Zuo J (2015). Preventing ototoxic hearing loss by inhibiting histone deacetylases. Cell Death Dis.

[55] Balogová Z, Popelář J, Chiumenti F, Chumak T, Burianová JS, Rybalko N, et al. Age-related differences in hearing function and cochlear morphology between male and female Fischer 344 rats. Front Aging Neurosci. 2018;9:428. http://journal.frontiersin.org/article/10.3389/fnagi.2017.00428/full. 10.3389/fnagi.2017.00428 PMC5758597 29354051

[56] Henry KR (2004). Males lose hearing earlier in mouse models of late-onset age-related hearing loss; females lose hearing earlier in mouse models of early-onset hearing loss. Hear Res.

[57] Ohlemiller KK, Dahl AR, Gagnon PM (2010). Divergent aging characteristics in CBA/J and CBA/CaJ mouse cochleae. J Assoc Res Otolaryngol.

[58] Hirose K, Liberman MC (2003). Lateral wall histopathology and endocochlear potential in the noise-damaged mouse cochlea. JARO J Assoc Res Otolaryngol.

[59] Wang Y, Hirose K, Liberman MC (2002). Dynamics of noise-induced cellular injury and repair in the mouse cochlea. J Assoc Res Otolaryngol.

[60] Caras ML. Estrogenic modulation of auditory processing: a vertebrate comparison. Front. Neuroendocrinol. San Diego, Academic Press INC Elsevier Science; 2013;34:285–299. 10.1016/j.yfrne.2013.07.006 PMC3788044 23911849

[61] Charitidi K, Meltser I, Canlon B (2012). Estradiol treatment and hormonal fluctuations during the estrous cycle modulate the expression of estrogen receptors in the auditory system and the prepulse inhibition of acoustic startle response. Endocrinology.

[62] Simonoska R, Stenberg A, Masironi B, Sahlin L, Hultcrantz M (2009). Estrogen receptors in the inner ear during different stages of pregnancy and development in the rat. Acta Otolaryngol.

[63] Stenberg AE, Wang H, Fish J, Schrott-Fisher A, Sahlin L, Hultcrantz M (2001). Estrogen receptors in the normal adult and developing human inner ear and in Turner’s syndrome. Hear Res.

[64] Motohashi R, Takumida M, Shimizu A, Konomi U, Fujita K, Hirakawa K (2010). Effects of age and sex on the expression of estrogen receptor α and β in the mouse inner ear. Acta Otolaryngol.

[65] Meltser I, Tahera Y, Simpson E, Hultcrantz M, Charitidi K, Gustafsson J-A (2008). Estrogen receptor beta protects against acoustic trauma in mice. J Clin Invest.

[66] Simonoska R, Stenberg AE, Duan M, Yakimchuk K, Fridberger A, Sahlin L (2009). Inner ear pathology and loss of hearing in estrogen receptor-beta deficient mice. J Endocrinol.

[67] Willott JF (2009). Effects of sex, gonadal hormones, and augmented acoustic environments on sensorineural hearing loss and the central auditory system: insights from research on C57BL/6J mice. Hear Res.

