# Supplementary figures and images for: The impact of biological sex on the response to noise and otoprotective therapies against acoustic injury in mice
Source: Biol Sex Differ. 2018 Mar 12;9:12. doi: 10.1186/s13293-018-0171-0 (PMC5848513; doi:10.1186/s13293-018-0171-0)

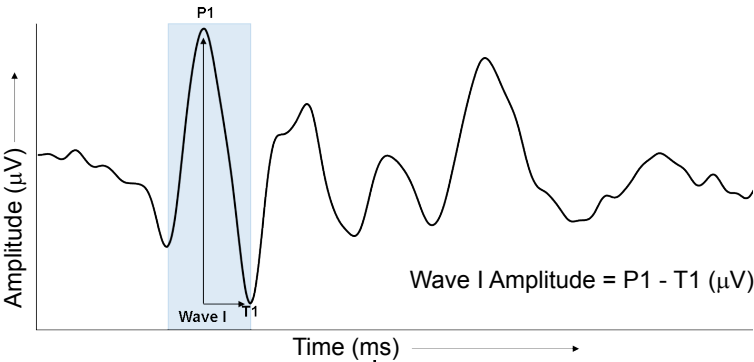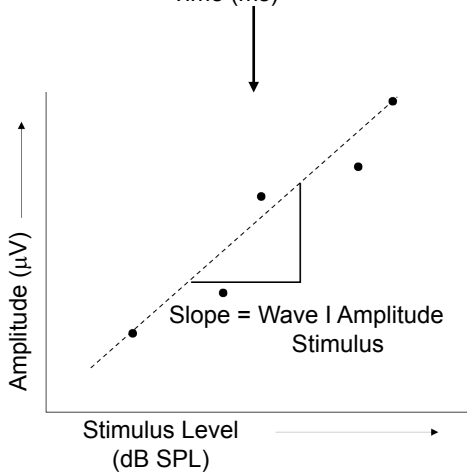

Supplement: Supplementary file 1 — Schematic showing ABR wave I extraction and analysis. Peak (P1) and trough (T1) values of wave I of the ABR traces (wave shaded in blue) were automatically extracted at stimuli levels from 55 to 85 dB SPL using a MatLab script. Wave I amplitudes were then plotted as a function of the stimuli levels. SigmaPlot was used to perform linear regression (dotted line) and calculate the slope (solid lines). Slopes were then compared between the different groups at 16 kHz. (PDF 471 kb) [file 13293_2018_171_MOESM1_ESM.pdf]

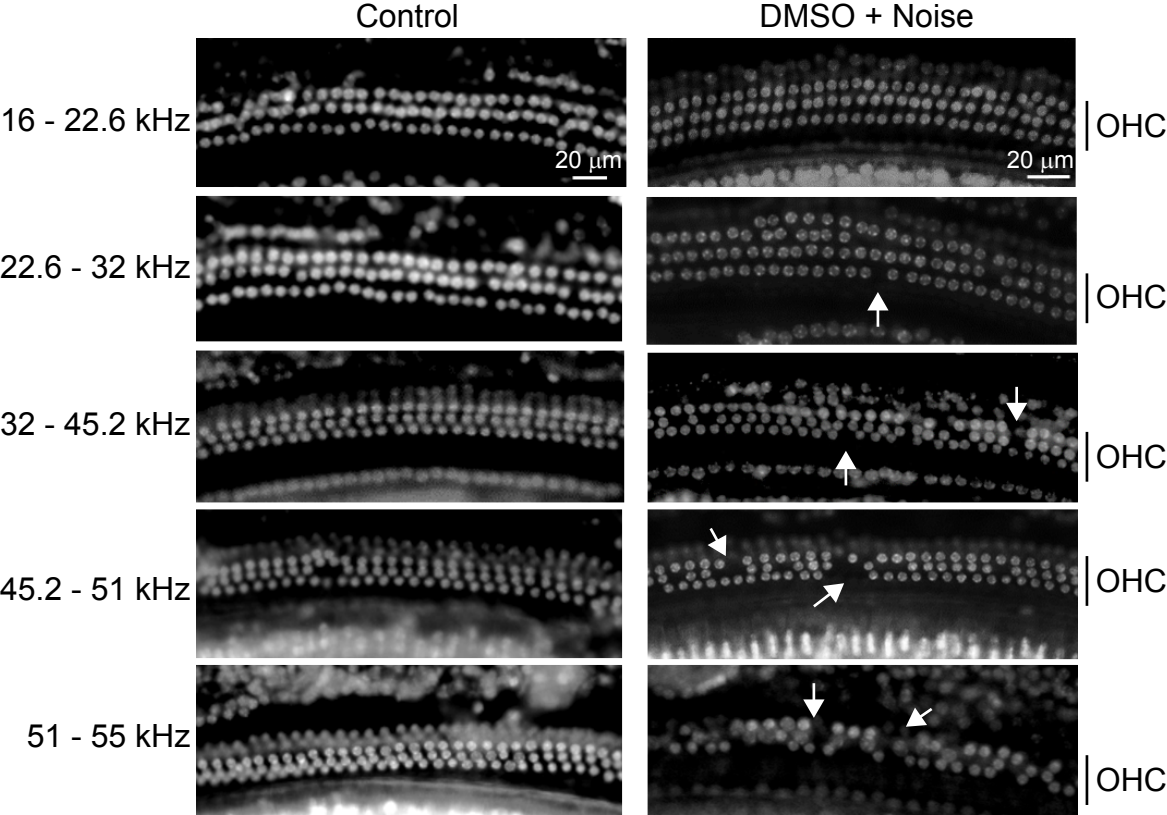

Supplement: Supplementary file 3 — OHC loss along the cochlear duct. Representative fluorescence microscopy images of the Organ of Corti at the level of the OHC (counter-stained with DAPI) at different frequency bands from controls and mice exposed at 101 dB SPL. There is little to no OHC loss in the control animals, whereas extensive OHC loss is seen above 32 kHz in animals exposed to noise. Scale bar represents 20 μm. (PDF 1120 kb) [file 13293_2018_171_MOESM3_ESM.pdf]

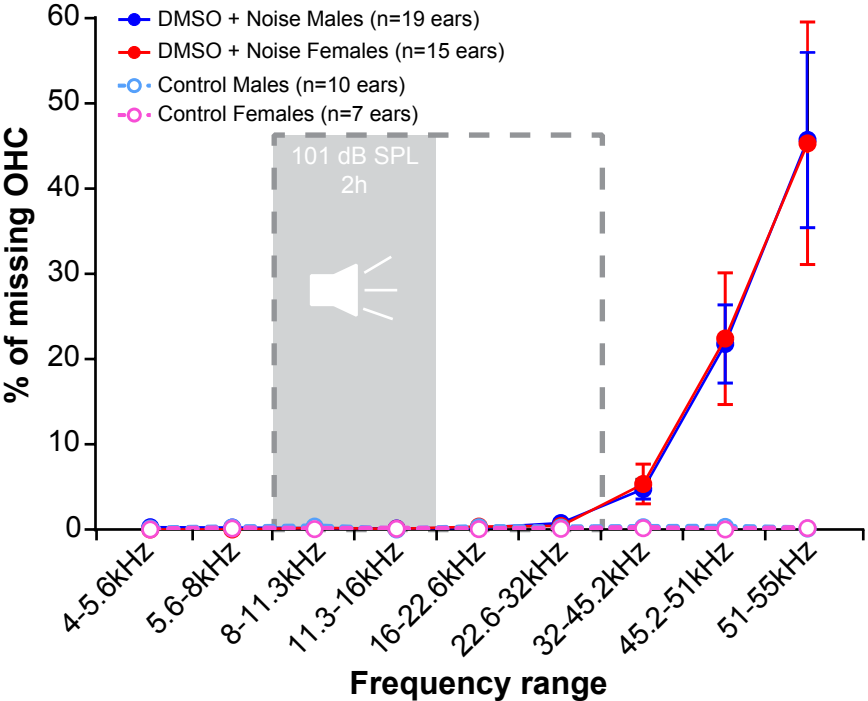

Supplement: Supplementary file 4 — OHC loss does not account for the frequency-specific PTS at 16 and 24 kHz or the sex differences in NIHL. Line graph indicating the percentage of OHC loss from apex to base in vehicle-treated noise-exposed animals compared to control non-noise-exposed animals. The frequency range of noise exposure is shaded gray and a gray dotted line outlines the frequency range where significant PTS is seen. Error bars indicate S.E.M. (PDF 396 kb) [file 13293_2018_171_MOESM4_ESM.pdf]

**16 kHz**

Control

DMSO + Noise

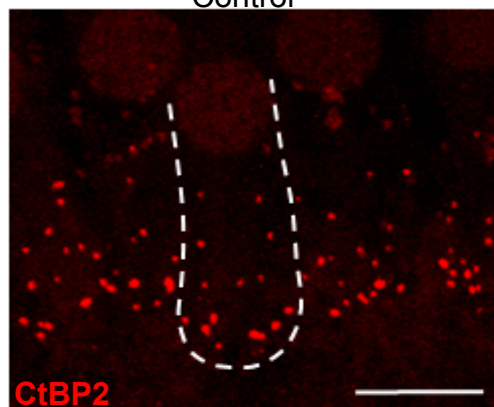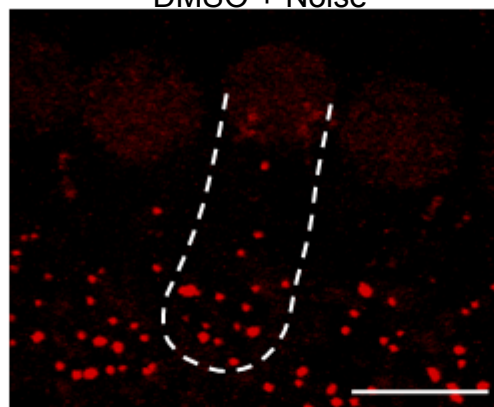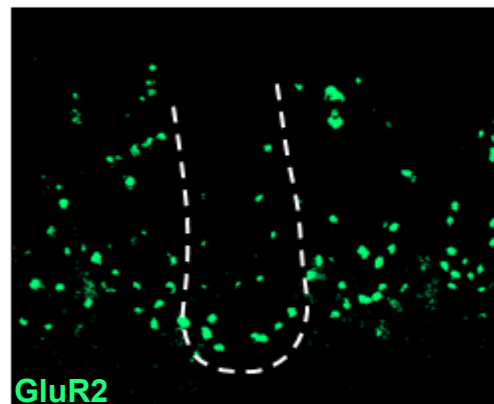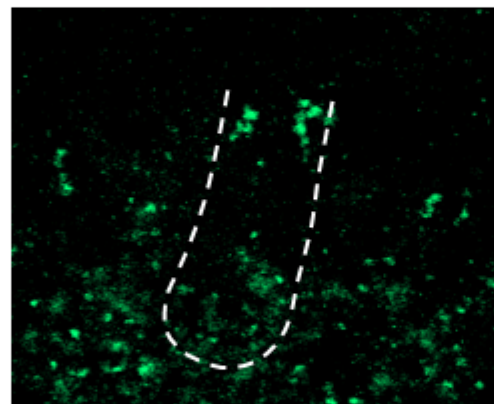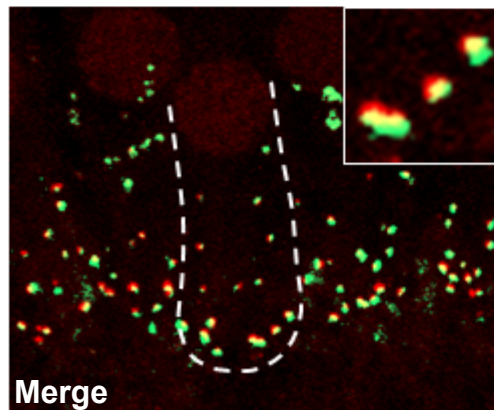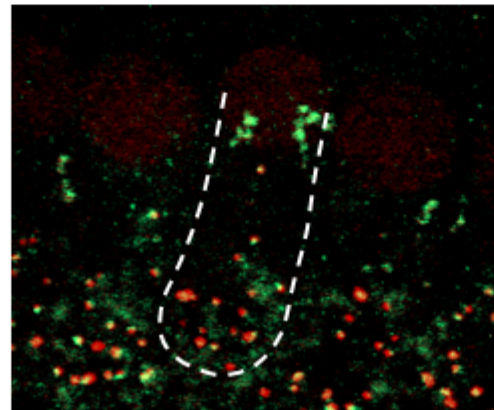**24 kHz**

Control

DMSO + Noise

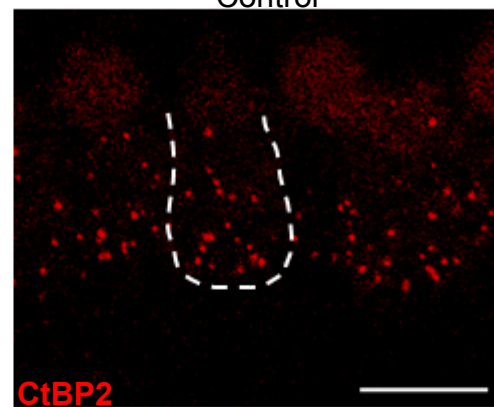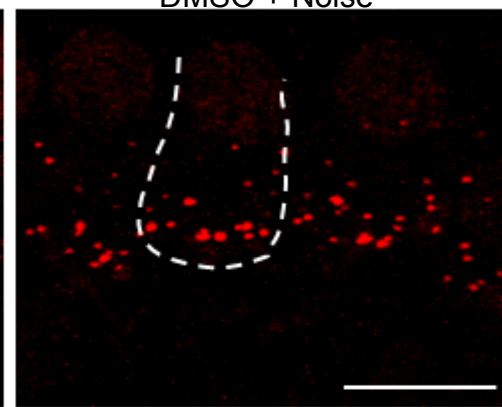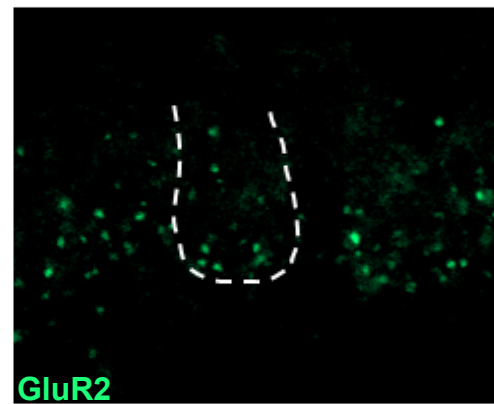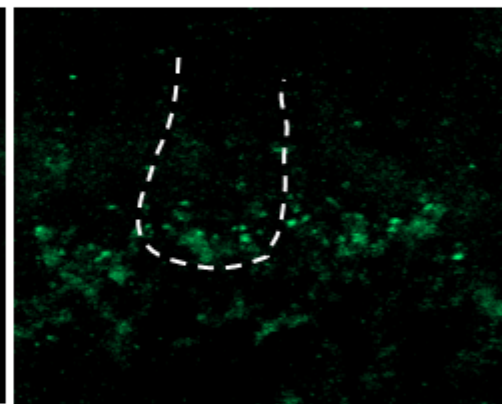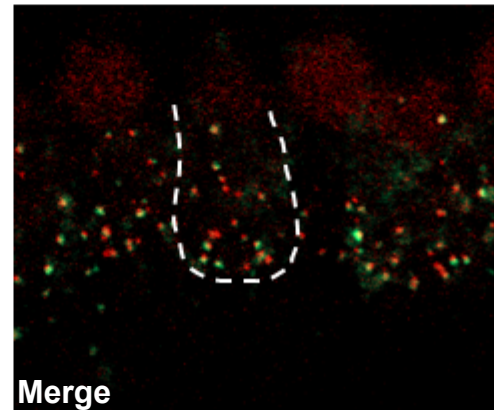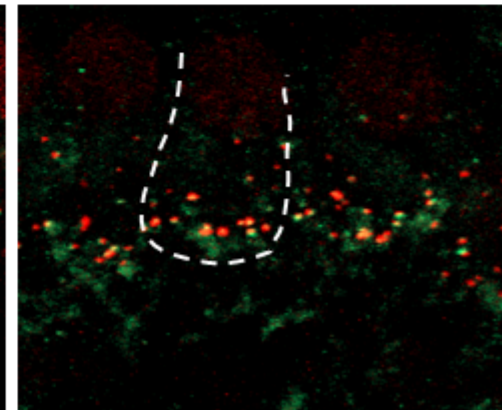

Supplement: Supplementary file 5 — Pre-synaptic ribbons and active synapses at 16 kHz and 24 kHz. Representative fluorescence microscopy images of IHC stained for CtBP2 (red) and GluR2 (green) at 16 kHz (left) and 24 kHz (right) from control and noise-exposed mice. The dotted lines represent the approximate border of one IHC. The inset in the bottom left corner image represent a zoom in of active synapses where CtBP2 and GluR2 partially co-localize. Scale bar represents 10 μm. (PDF 1242 kb) [file 13293_2018_171_MOESM5_ESM.pdf]

**a**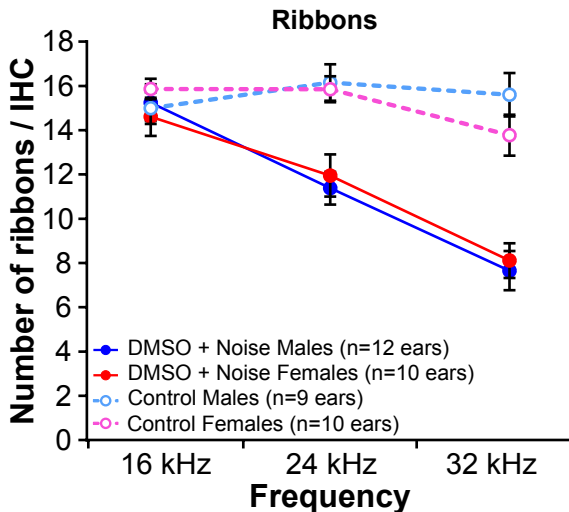**b**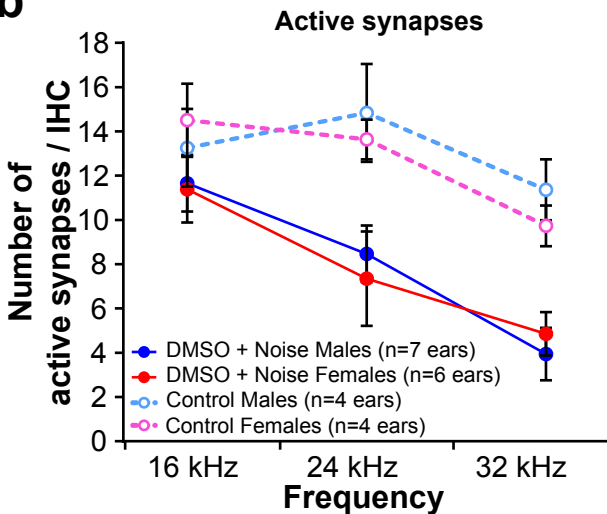

Supplement: Supplementary file 6 — Effect of noise on pre-synaptic ribbons and active synapses in IHC. Graphs representing the number of pre-synaptic ribbons (a) and active synapses (b) in IHC of control and vehicle-treated noise-exposed animals. A significant decrease in pre-synaptic ribbons and active synapses is observed at 24 and 32 kHz in both males and females, but no difference is seen between sexes. Error bars indicate S.E.M. (PDF 418 kb) [file 13293_2018_171_MOESM6_ESM.pdf]
