# Supplementary material for: The impact of biological sex on the response to noise and otoprotective therapies against acoustic injury in mice
Source: Biol Sex Differ. 2018 Mar 12;9:12. doi: 10.1186/s13293-018-0171-0 (PMC5848513; doi:10.1186/s13293-018-0171-0)
Supplement: Supplementary file 2 — Table S1.Comparison of average hearing thresholds at baseline in male and female mice (Sidak’s multiple comparison test; ns: non-significant). Table S2. Average threshold shift values in dB at 24 h post-noise exposure (CTS) and 15 days post-noise exposure (PTS) in vehicle-treated males and females (Tukey’s multiple comparisons test). Table S3. Statistical values for interactions between the two factors following a two-way ANOVA. The degrees of freedom for the numerator (DFn) and denominator (DFd) are shown in parenthesis before the F value. Significant results are shown in bold font. Table S4. Comparison of average ABR thresholds shifts at 24 h (CTS) and 15 days (PTS) post-noise exposure in male and female mice treated with vehicle only (Sidak’s multiple comparison test; ns: non-significant). Table S5. Values for the percentage of OHC loss within 32–45.2 kHz, 45.2–51 kHz, and 51–55 kHz. Progressive OHC loss is seen up to 55 kHz which is the highest frequency counted. Both male and female animals show a similar pattern of OHC loss. ± represent S.E.M. (unpaired t test to compare male and female mice). Table S6. Comparison of average threshold shift values in dB at 24 h post-noise exposure (CTS) and 15 days post-noise exposure (PTS) between vehicle- and SAHA-treated males and females separately (Sidak’s multiple comparisons test; ns: non-significant). Table S7. Comparison of average threshold shift values in dB at 24 h post-noise exposure (CTS) and 15 days post-noise exposure (PTS) between vehicle- and SAHA-treated animals (Sidak’s multiple comparisons test; ns: non-significant). (PDF 257 kb) [file 13293_2018_171_MOESM2_ESM.pdf]

Table S1. Comparison of average hearing thresholds at baseline in male and female mice. Significant results are shown in bold font (Sidak's multiple comparison test; ns: non-significant).

	**Hearing Thresholds at baseline**			
	**8 kHz**	**16 kHz**	**24 kHz**	**32 kHz**
**Males**	8.7	1.6	15.7	27.6
**Females**	9.7	2.1	14.3	24.9
***p* value**	ns	ns	ns	**0.0008**

Table S2. Average threshold shift values in dB at 24 h post noise exposure (CTS) and 15 days post noise exposure (PTS) in vehicle treated males and females. Significant results are shown in bold font (Tukey's multiple comparisons test).

		**8 kHz**	***p* value**	**16 kHz**	***p* value**	**24 kHz**	***p* value**	**32 kHz**	***p* value**
**CTS**	Both Sexes	9.9	**<0.0001**	39.4	**<0.0001**	36.1	**<0.0001**	33.0	**<0.0001**
	Males	12.9	**<0.0001**	46.7	**<0.0001**	44.0	**<0.0001**	34.6	**<0.0001**
	Females	7.1	**0.0004**	32.5	**<0.0001**	28.8	**<0.0001**	31.4	**<0.0001**
**PTS**	Both Sexes	10.5	**<0.0001**	32.7	**<0.0001**	23.6	**<0.0001**	10.4	**<0.0001**
	Males	11.9	**<0.0001**	39.8	**<0.0001**	32.9	**<0.0001**	15.8	**<0.0001**
	Females	9.1	**<0.0001**	26.1	**<0.0001**	15.0	**<0.0001**	5.4	**0.0103**

Table S3. Statistical values for interactions between the two factors following a two-way ANOVA. The degrees of freedom for the numerator (DFn) and denominator (DFd) are shown in parenthesis before the F value. Significant results are shown in bold font.

				**F(DFn, DFd)**	***p* value**
**Frequency x Time**	Figure 1	ABR Thresholds		**(9, 752) 23.56**	**< 0.0001**
**Frequency x Sex**	Figure 3	CTS		**(3, 208) 4.3**	**0.006**
		PTS		**(3, 208) 4.01**	**0.008**
**Sex x Treatment (SAHA)**	Figure 7	8 kHz	CTS	(1, 104) 0.299	0.585
			PTS	(1, 102) 0.038	0.845
		16 kHz	CTS	(1, 104) 0.005	0.942
			PTS	(1, 102) 0.567	0.453
		24 kHz	CTS	**(1, 104) 8.36**	**0.005**
			PTS	(1, 102) 1.653	0.201
		32 kHz	CTS	**(1, 104) 4.667**	**0.033**
			PTS	(1, 102) 2.286	0.134
**Treatment (SAHA) x Frequency**	Figure 8	CTS		(3, 424) 2.177	0.090
		PTS		(3, 416) 1.067	0.363

Table S4. Comparison of average ABR thresholds shifts at 24h (CTS) and 15 days (PTS) post noise-exposure in male and female mice treated with vehicle only. Significant results are shown in bold font (Sidak's multiple comparison test; ns: non-significant).

		**8 kHz**	**16 kHz**	**24 kHz**	**32 kHz**
**CTS**	Males	12.9	46.7	44.0	34.6
	Females	7.1	32.5	28.8	31.4
	p value	ns	**< 0.0001**	**< 0.0001**	ns
**PTS**	Males	11.9	39.8	32.9	15.8
	Females	9.1	26.1	15.0	5.4
	p value	ns	**< 0.0001**	**< 0.0001**	**0.005**

Table S5. Values for the percentage of OHC loss within 32-45.2 kHz, 45.2-51 kHz and 51-55 kHz. Progressive OHC loss is seen up to 55 kHz which is the highest frequency counted. Both male and female animals show a similar pattern of OHC loss. ± represent S.E.M. (Unpaired *t* test to compare male and female mice).

	**Frequency range**								
	**32-45.2kHz**			**45.2-51kHz**			**51-55kHz**		
	Males	Females	*p* value	Males	Females	*p* value	Males	Females	*p* value
**Controls**	0.29 ± 0.10	0.18 ± 0.12	0.45	0.34 ± 0.34	0.00 ± 0.00	0.34	0.15 ± 0.15	0.18 ± 0.18	0.93
**DMSO + Noise**	4.76 ± 1.17	5.36 ± 2.32	0.80	21.8 ± 4.57	22.4 ± 7.73	0.94	45.7 ± 10.3	45.3 ± 14.2	0.98

Table S6. Comparison of average threshold shift values in dB at 24h post noise exposure (CTS) and 15 days post noise exposure (PTS) between vehicle and SAHA treated males and females separately. Significant results are shown in bold font (Sidak's multiple comparisons test; ns: non-significant).

		**8 kHz**			**16 kHz**			**24 kHz**			**32 kHz**		
		Vehicle	SAHA	*p* value	Vehicle	SAHA	*p* value	Vehicle	SAHA	*p* value	Vehicle	SAHA	*p* value
**CTS**	Males	12.9	9.6	ns	46.7	38.4	ns	44.0	31.1	**0.0006**	34.6	31.2	ns
	Females	7.1	5.2	ns	32.5	24.6	**0.04**	28.8	30.0	ns	31.4	36.0	ns
**PTS**	Males	11.9	8.7	ns	39.8	34.0	ns	32.9	20.4	**0.002**	15.8	8.3	ns
	Females	9.1	5.4	ns	26.1	15.6	**0.003**	15.0	9.0	ns	5.4	3.8	ns

Table S7. Comparison of average threshold shift values in dB at 24h post noise exposure (CTS) and 15 days post noise exposure (PTS) between vehicle and SAHA treated animals. Significant results are shown in bold font (Sidak's multiple comparisons test; ns: non-significant).

	8 kHz			16 kHz			24 kHz			32 kHz		
	Vehicle	SAHA	*p* value	Vehicle	SAHA	*p* value	Vehicle	SAHA	*p* value	Vehicle	SAHA	*p* value
**CTS**	9.9	7.5	ns	39.4	31.8	**0.007**	36.1	30.6	ns	33.0	33.5	ns
**PTS**	10.5	7.0	ns	32.7	24.8	**0.009**	23.6	14.7	**0.002**	10.4	6.1	ns
